# Somatic Mitochondrial DNA Mutations in Diffuse Large B-Cell Lymphoma

**DOI:** 10.1038/s41598-018-21844-6

**Published:** 2018-02-26

**Authors:** Andy G. X. Zeng, Andy C. Y. Leung, Angela R. Brooks-Wilson

**Affiliations:** 1 Canada’s Michael Smith Genome Sciences Centre, BC Cancer Agency, Vancouver, British Columbia Canada; 2 Department of Statistics, University of British Columbia, Vancouver, British Columbia Canada; 3 Department of Biomedical Physiology and Kinesiology, Simon Fraser University, Burnaby, British Columbia Canada

## Abstract

Diffuse Large B-Cell Lymphoma (DLBCL) is an aggressive hematological cancer for which mitochondrial metabolism may play an important role. Mitochondrial DNA (mtDNA) encodes crucial mitochondrial proteins, yet the relationship between mtDNA and DLBCL remains unclear. We analyzed the functional consequences and mutational spectra of mtDNA somatic mutations and private constitutional variants in 40 DLBCL tumour-normal pairs. While private constitutional variants occurred frequently in the D-Loop, somatic mutations were randomly distributed across the mitochondrial genome. Heteroplasmic constitutional variants showed a trend towards loss of heteroplasmy in the corresponding tumour regardless of whether the reference or variant allele was being lost, suggesting that these variants are selectively neutral. The mtDNA mutational spectrum showed minimal support for ROS damage and revealed strand asymmetry with increased C > T and A > G transitions on the heavy strand, consistent with a replication-associated mode of mutagenesis. These heavy strand transitions carried higher proportions of amino acid changes – which were also more pathogenic – than equivalent substitutions on the light strand. Taken together, endogenous replication-associated events underlie mtDNA mutagenesis in DLBCL and preferentially generate functionally consequential mutations. Yet mtDNA somatic mutations remain selectively neutral, suggesting that mtDNA-encoded mitochondrial functions may not play an important role in DLBCL.

## Introduction

Diffuse Large B-Cell Lymphoma (DLBCL) is the most common type of non-Hodgkin lymphoma (NHL). An aggressive and heterogeneous cancer, DLBCL can be categorized into multiple subtypes. The Cell of Origin (COO) classification system defines a germinal center B-cell type and an activated B-cell type and has prognostic value^1^. The consensus cluster classification (CCC) of molecular characteristics defines three subgroups: an oxidative phosphorylation (OxPhos) group, a B-cell receptor/proliferation group and a host response group^2^. The fact that altered expression of genes involved in oxidative phosphorylation occurs frequently enough to constitute a subgroup suggests that mitochondrial metabolism may play an important role in DLBCL^2–4^.

The mitochondrial genome plays a crucial role in cellular metabolism. Each mitochondrion within the cell has two to ten copies of the mitochondrial genome. At 16,569 base pairs in length, it encodes 13 key subunits within OxPhos complexes I, III, IV and V. Given its unique genetic code, the mitochondrial genome also contains its own translational machinery comprising 22 tRNAs and 2 rRNAs. Mitochondrial DNA (mtDNA) is estimated to have a ten-fold greater mutation rate than nuclear DNA^5^, which has commonly been attributed to its lower DNA repair efficiency and greater exposure to OxPhos generated reactive oxygen species (ROS)^6,7^. Mutated mtDNA molecules can be propagated by selection or genetic drift, ultimately constituting either a fraction of the mitochondrial genomes (heteroplasmy) or all of the mitochondrial genomes (homoplasmy) within a cell^7^.

ROS overproduction arising from deleterious mutations in OxPhos complexes has been proposed as a primary link between mtDNA and carcinogenesis in many cancers^8–10^. A ROS-mediated relationship may also apply with mtDNA and B-Cell Lymphoma. In PolgA mutator mouse models, homozygous PolgA mutants displayed a 3 to 5 fold increase in mtDNA point mutations relative to wild-type PolgA siblings from the heterozygous parents. This was accompanied by reduced cytochrome c oxidase activity, increased ROS production and increased risk of lymphoid tumour development^11^. Another strain carrying a specific mtDNA mutation that impaired complex I activity and induced ROS overproduction also demonstrated higher risk of developing B-cell lymphoma^12^. Administration of a ROS scavenger in these same mice reduced ROS levels in the bone marrow and prevented lymphoma development, further supporting a link between mtDNA, ROS and B-cell lymphoma^13^.

Despite these connections between mtDNA and B-Cell Lymphoma, the mutational landscape of the mitochondrial genome in lymphoma remains unclear. To our knowledge, mitochondrial genomes from only four lymphoma samples were analyzed as part of a study encompassing 31 cancer types^14^. The Cancer Genome Characterization Initiative conducted whole genome sequencing (WGS) of 40 tumour and peripheral blood (normal) pairs to characterize the mutational landscape in the nuclear genome of DLBCL^15^. We accessed this data through the NCBI database of Genotypes and Phenotypes (dbGaP), extracted mtDNA information and characterized the somatic mutations and constitutional variants in the mitochondrial genomes of the 40 DLBCL tumour-normal pairs.

## Results

### Characterization of Somatic Mutations and Private Constitutional Variants

We successfully extracted mitochondrial reads for 39 of the 40 tumour-normal pairs in our analysis; one pair was eliminated due to the absence of mitochondrial reads in the normal sample. One variant, C12705T, was present as a heteroplasmy in 26 samples. Inspection of the variant with the Integrative Genome Viewer^16^ showed that all reads containing C12705T also contained another variant, G12684A, which otherwise did not appear at that locus. These two variants were flagged as potential artefacts and, after confirmation of their absence in corresponding RNA-seq data, were excluded from further analysis.

The average depth, number of total variants, number of unique variants and number of samples with one or more of each type of variant are shown in Table 1. A complete list of the somatic mutations can be found in Supplementary Table S1. Using a VAF threshold of 0.02, all samples carried constitutional variants, 34 (87%) samples carried at least one private constitutional variant, 23 (59%) carried at least one constitutional heteroplasmy and 29 (74%) had at least one somatic mutation in the tumour. The average read depth among called variants was around 2000× for all variant types. Of the 34 patients carrying private constitutional variants, the number of private constitutional variants per sample ranged from 1 to 7, with a mean of 3 variants per sample. Of the 29 patients carrying somatic mutations, the number of somatic mutations per sample ranged from 1 to 5, with a mean of 2 mutations per sample.

**Table 1:** Summary of mitochondrial variants in DLBCL sample set.

Sample Summary	Total Constitutional	Private Constitutional	Heteroplasmic Constitutional	Somatic Mutation
Avg Depth ± Stdev	2205 ± 1227	2237 ± 1203	2081 ± 1268	1990 ± 977
Total Variants	1006	108	40	60
Unique Variants	349	99	38	59
# of Samples	39	34	23	29

Avg Depth ± Stdev = Average read depth and standard deviation of all variants; # of Samples = Number of samples containing one of more variant.

**Figure 1:**
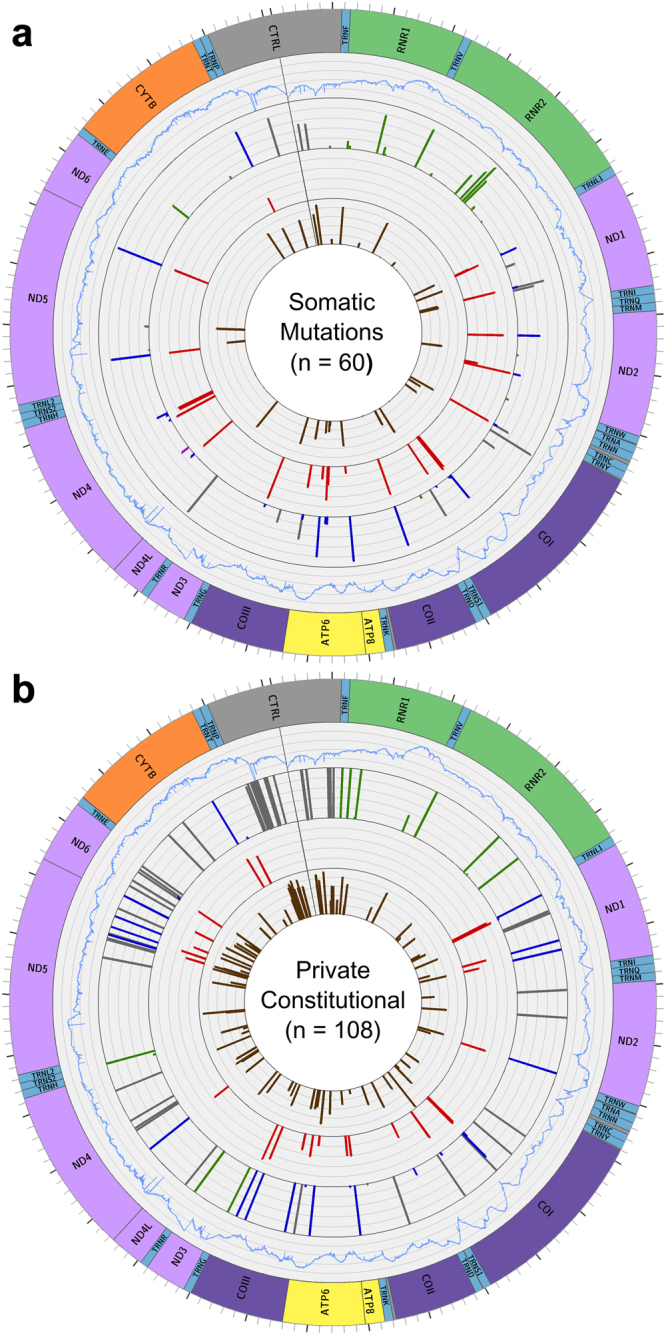
mtDNA variants in DLBCL. Graphs from periphery to center: (1) Map of the mitochondrial genome (2) Average sequence depth for each position among the samples carrying variants, ranging from 0 to 4000 reads. (3) SNVs found in DLBCL, with the height of each bar depicting the VAF. tRNA and rRNA variants are green, stop-gains are red, non-synonymous variants are blue, synonymous and non-coding variants are grey. (4) The MutPred pathogenicity score for each non-synonymous variant. (5) The log GenBank frequency of each variant, represented as 5 + log10(x/30589), where x is the number of genomes in GenBank that carry that variant. (**a**) Somatic Mutations in 29 samples. (**b**) Private constitutional variants in 34 samples.

From each of the 108 private constitutional variants and 60 somatic mutations, the VAF was determined; private constitutional variants were predominantly present as homoplasmies or low-level heteroplasmies (VAF < 10%), while somatic mutations were exclusively heteroplasmic and showed more variation in VAF (Fig. 1). The frequency of each variant within 30589 mtDNA sequences (normal and diseased) from GenBank showed that in comparison to private constitutional variants, somatic mutations had a significantly lower average GenBank frequency (p = 0.0043, t = −2.898). Notably, 52% of somatic mutations had not been previously reported in GenBank while only 7% of private constitutional variants were novel (Fig. 1).

We also assessed the distribution of private constitutional variants and somatic mutations by dividing the mitochondrial genome into seven functional regions: non-coding (all non-coding regions, including the D-Loop), rRNA (both rRNA-coding genes), tRNA (all tRNA-coding genes), OxPhos complex I (ND1, ND2, ND3, ND4, ND4L, ND5, ND6), complex III (CYB), complex IV (COI, COII, COIII) and complex V (ATP6, ATP8). The proportion of total variants within each region, as well as the percentage of the genome (by length) that each region constituted, is shown in Table 2. Private constitutional variants were distributed in a pattern distinct from that expected by chance (p = 7.81e-13, *χ*^2^ = 68.628) with a higher proportion of variants in the D-Loop, while the distribution of somatic mutations across the mitochondrial genome did not differ significantly from chance. Recurrent mutations were observed in both groups of variants: 8 positions had recurrent private constitutional variants (T146C, T204C, G709A, G1719A, A2905G, G12236A, G16129A, T16311C), while the only recurrent somatic mutation was G11711A, a non-synonymous variant in ND4 (complex I) that appeared as a low-level heteroplasmy in two samples.

**Table 2:** Variant distribution across mtDNA functional regions.

Region	Genomic Length (bp)	Somatic Mutation	Private Constitutional
Non-Coding	1153 (7%)	4 (7%)	30 (28%)
rRNA	2513 (15%)	13 (22%)	12 (11%)
tRNA	1508 (9%)	3 (5%)	7 (6%)
Complex I	6356 (38%)	21 (35%)	35 (32%)
Complex III	1141 (7%)	2 (3%)	4 (4%)
Complex IV	3010 (18%)	13 (22%)	15 (14%)
Complex V	888 (5%)	4 (7%)	5 (5%)
Total	16519	60	108

Of the 40 somatic mutations observed in protein-coding regions, 13 (33%) were synonymous while 26 (65%) were non-synonymous and one was a stop-gain. In contrast, of the 59 private constitutional variants observed in protein-coding regions, 34 (58%) were synonymous while 25 (42%) were non-synonymous. The frequency of somatic mutations occurring across the three different codon positions was relatively uniform (35%, 27.5%, 37.5%, in order of position within the codon) in comparison to that of private constitutional variants (27%, 15%, 58%), which was notably skewed toward changes in the third (wobble) position, in which changes are mostly synonymous. Pathogenicity scores were obtained for non-synonymous variants using Mutpred^17^: Somatic mutations had a higher median pathogenicity score (0.72, on a scale of 0–1) than private constitutional variants (0.49). Sequence depth, VAF, GenBank frequency, variant distribution, variant function and pathogenicity of somatic mutations and constitutional variants were visualized using Circos^18^ (Fig. 1).

To determine whether mtDNA mutations influence the clinical manifestation of DLBCL, we considered each patient’s tumor size, lactate dehydrogenase ratio, number of extra-nodal sites, performance status, international prognostic index, treatment status and patient response to treatment. We found no significant association between mtDNA mutational burden and any of these clinical phenotypes.

### Heteroplasmic Shifts

**Figure 2:**
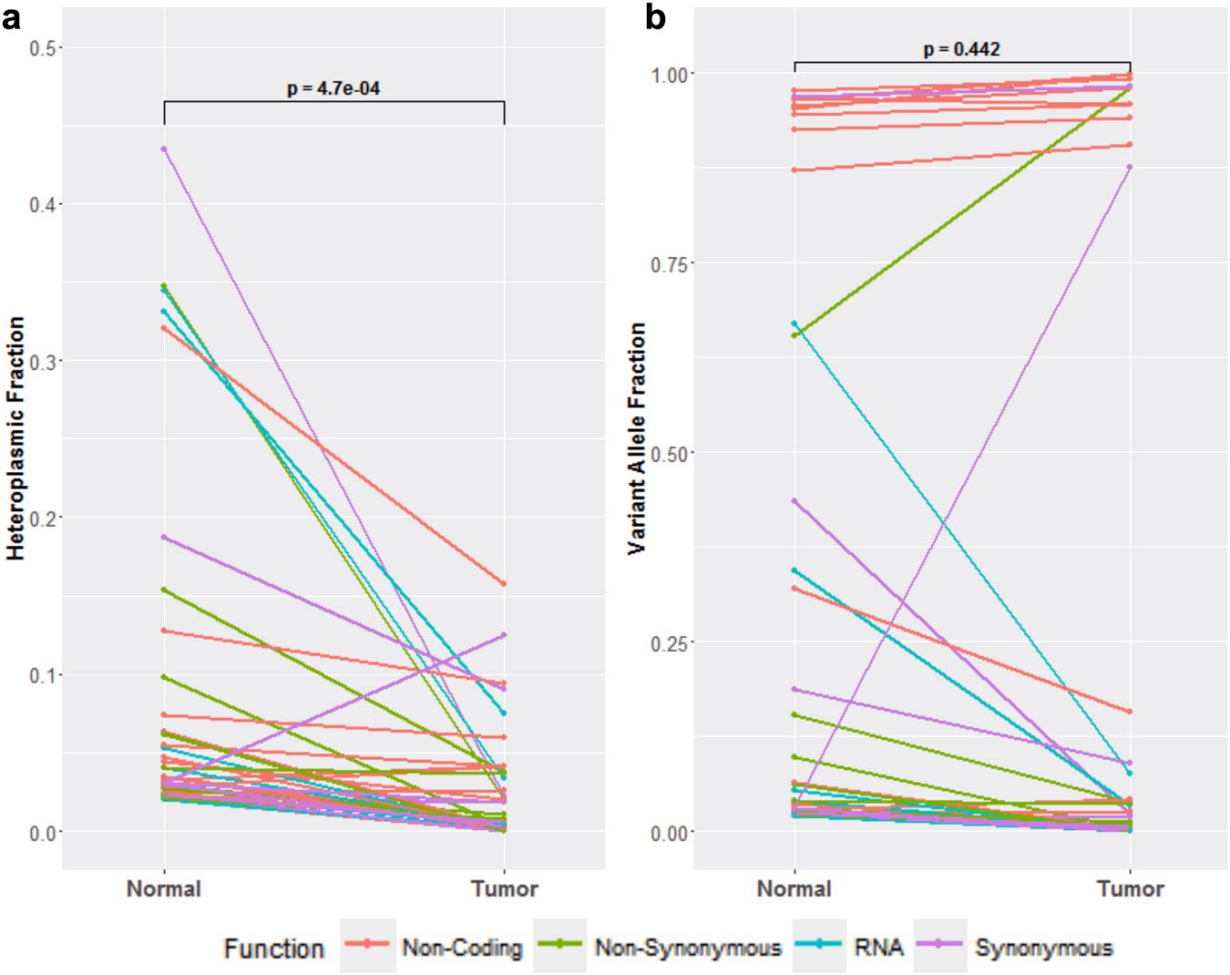
Shifts in heteroplasmic variant frequencies through lymphomagenesis. Constitutional variants identified as heteroplasmic in the normal tissue were compared to their counterpart in the matched tumour tissue. p values were generated by a paired t-test. (**a**) Changes in heteroplasmic fraction. (**b**) Changes in variant allele fraction.

Heteroplasmic mtDNA alleles in normal tissue are expected to experience shifts in allele proportions through lymphomagenesis. To track such changes, the heteroplasmic fractions (HF, minor allele count/total read count) were determined for each of the heteroplasmic constitutional alleles and their corresponding positions in the matched tumour sample (Fig. 2a). Heteroplasmic constitutional alleles showed a significant trend towards reduction or loss of the minor allele – represented as decreases in HF – in the corresponding tumour (p = 0.00047, t = 3.815).

To understand whether these heteroplasmic shifts through lymphomagenesis are driven by selective or stochastic processes, we determined variant allele fractions (VAF, variant allele count/total read count) for each of the heteroplasmic constitutional variants and their corresponding positions in the matched tumour sample (Fig. 2b). Unlike the HF trend, VAF changes were more varied and neither variant nor reference alleles were preferentially fixated or lost in the corresponding tumour.

### Mutational Spectrum

**Figure 3:**
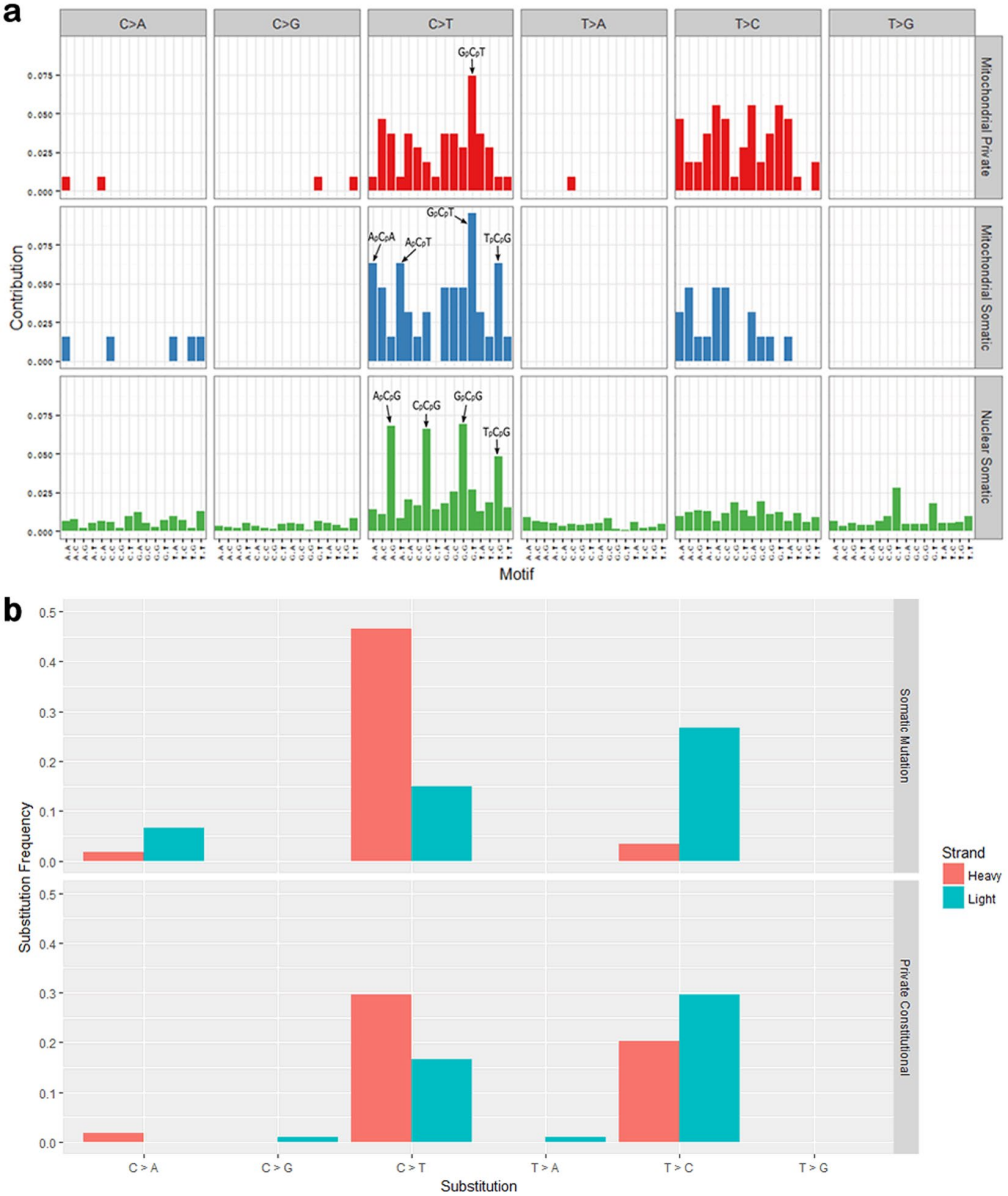
Mutational spectra of mtDNA variants in DLBCL. (**a**) Substitution frequencies by 96 trinucleotide context. 108 mitochondrial private constitutional variants, 60 mitochondrial somatic mutations and 3419 nuclear exonic somatic mutations are shown. (**b**) Frequency of substitution type for each mtDNA strand for somatic mutations and private constitutional variants.

To understand the processes driving the formation of private constitutional variants and somatic mutations, we used SomaticSignatures 19 to determine the substitution frequencies in the context of different 5′ and 3′ nucleotides flanking the mutation site and to generate mutational spectra for mtDNA private constitutional variants, mtDNA somatic mutations and nuclear exonic somatic mutations (Fig. 3a). C > T substitutions at CpG sites were present at the highest frequencies among nuclear somatic mutations but this was not the case with mtDNA variants; rather, the most frequently substituted site among both mtDNA somatic mtuations and private constitutional variants was GpCpT. Somatic mutations in mtDNA showed a modest elevation in the proportion of C > A substitutions – a signature of ROS damage^19^ –compared to mtDNA private constitutional variants (0.08 vs 0.02), but the difference was not statistically significant. Probing further into these mutational spectra, we determined the frequencies of different C and T substitutions occurring on the light strand (L-strand; forward rCRS sequence) and the heavy strand (H-strand; reverse complement of rCRS) (Fig. 3b). Among mitochondrial somatic mutations, there was asymmetry in the occurrence of C > T and T > C transitions for the two strands: C > T substitutions occurred primarily on the H-strand and T > C substitutions occurred primarily on the L-strand (these are equivalent to A > G on the H-strand). Notably, this strand asymmetry was more pronounced among somatic mutations than private constitutional variants.

**Figure 4:**
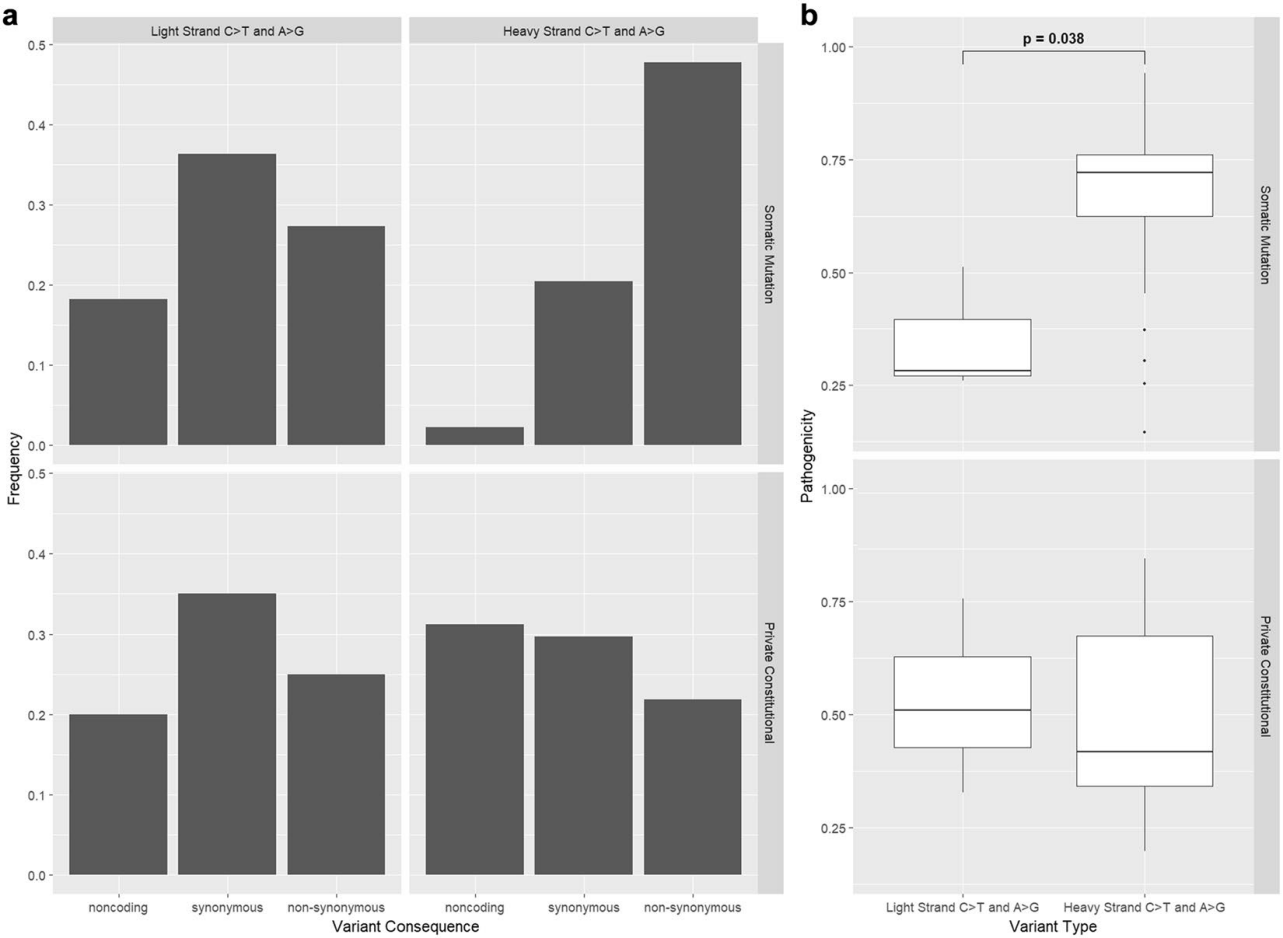
Consequences of C > T and A > G Variants on Light Strand vs Heavy Strand. (**a**) Frequency of Non-Coding, Synonymous and Non-Synonymous C > T and A > G variants by strand, for somatic mutations and private constitutional variants. (**b**) Pathogenicity of Non-Synonymous C > T and A > G variants by strand, for somatic mutations and private constitutional variants. p values were generated by a paired t-test.

To investigate this strand asymmetry, we compared C > T and A > G variants on the H-strand with C > T and A > G variants on the L-strand by assessing the frequencies of non-coding, synonymous and non-synonymous variants (Fig. 4a) as well as the pathogenicity of non-synonymous variants (Fig. 4b). Among somatic mutations, C > T and A > G variants on the H-strand included a lower proportion of non-coding variants and a higher proportion of non-synonymous variants than those on the L-strand. Among these non-synonymous variants, the pathogenicity was significantly higher for C > T and A > G variants on the H-strand compared to those on the L-strand. Neither of these results were reflected among private constitutional variants; instead, the proportion of non-coding C > T and A > G variants was modestly higher on the H-strand than the L-strand.

## Discussion

A study comparing somatic mutations and constitutional variants in mtDNA of acute myeloid leukemia (AML) and four other cancers from the Cancer Genome Atlas (TCGA) found non-synonymous changes in 86% of protein-coding somatic mutations but only 31% of protein-coding constitutional variants^20^. Non-synonymous changes among somatic mutations showed higher pathogenicity than those of constitutional variants, leading the authors to conclude that a higher burden of pathogenic mutations confers a selective advantage. In contrast, two studies using TCGA data^14,21^ demonstrated purifying selective pressure against deleterious and truncating somatic mtDNA mutations and did not observe any signs of positive selective pressures during tumourigenesis within the mitochondrial genome. Another study examining variants across multiple cancer types revealed non-random amino acid changes in cytochrome b and cytochrome c oxidase I, suggesting non-random mutations across specific regions of the mitochondrial genome^22^. McMahon *et al*. used TCGA data to compare somatic mutations and constitutional variants in 99 breast cancer patients and noted a high frequency of variants in the D-Loop: somatic mutations and constitutional variants were present at 2× and 4× higher frequencies, respectively, than expected by chance^23^.

We assessed the distribution of mtDNA variants across different functional regions of the mitochondrial genome (Table 2). In comparison to the variant distribution expected by chance, it is apparent that private constitutional variants were subject to purifying selection, which lowered the variant load in the coding regions relative to the D-Loop and consequently reduced the number of potentially pathogenic variants persisting in the genome. In contrast, the mtDNA somatic mutation distribution is similar to that expected by chance, suggesting that somatic mutations are selectively neutral in DLBCL. The trend towards loss of heteroplasmy (represented by HF, Fig. 2) is consistent with previous reports of a tendency towards allelic fixation in cancer cells^24^. The lack of directionality in VAF changes shows that the heteroplasmic variants in the peripheral blood favour neither the fixation or loss of the variant allele, suggesting that these heteroplasmic shifts occur through neutral drift rather than selection. These heteroplasmic variants are thus unlikely to confer fitness advantages. This is consistent with the results of Coller *et al*.^25^, who showed that fixation of heteroplasmies into homoplasmies can occur frequently in tumours in the absence of any selective pressures. Taken together, these results suggest that mtDNA somatic mutations are tolerated by DLBCL tumours and have minimal influence on their biological and clinical characteristics.

In interpreting the mutational spectrum, we considered that 8-oxo-G damage during library preparation may introduce artefactual C:G > A:T substitutions that confound mutational spectrum interpretation. These variants are also key indicators of ROS-mediated DNA damage^26^, highlighting the importance of distinguishing between artefactual and biological G:G > A:T substitutions in order to understand the origin of mtDNA somatic mutations. These library preparation artefacts primarily occur within the CCG > CAG sequence context^27^ and were detected at VAFs ranging from 0.01–0.05 in many studies of nuclear DNA^28^. However, given the higher copy number of mtDNA compared to nuclear DNA, a single 8-oxo-G insult at any position during library preparation would affect a relatively lower proportion of total mtDNA and the sequencing C:G > A:T artefact should manifest at a lower VAF than reported in nuclear DNA sequences. This is illustrated by a study of mtDNA variants present below 0.01 VAF, in which artefactual C:G > A:T substitutions dominated the mutational spectrum^29^.

In contrast, we report that C:G > A:T substitutions ranged in VAF from 0.04–0.983 and only represented 2% of private constitutional variants and 8% of mtDNA somatic mutations, with none of these substitutions occurring within a CCG > CAG sequence context. Given that tumor and normal mtDNA undergo similar library preparation protocols prior to sequencing, the higher proportion of C:G > A:T substitutions observed in somatic mutations compared to private constitutional variants is likely to be independent of library preparation artefacts, but this difference is ultimately not statistically significant and constitutes only a small proportion of somatic mutations in DLBCL. This suggests that ROS has a limited role in mtDNA mutagenesis and is consistent with the general lack of ROS-mediated DNA damage signatures in mtDNA somatic mutations reported by Kauppila and Stewart^30^ and Itsara *et al*.^31^.

There were clear distinctions between the mutational spectra of nuclear exonic somatic mutations and mtDNA somatic mutations (Fig. 3a). In particular, nuclear exonic mutations included a high frequency of C > T substitutions at C_p_G sites while mtDNA mutations did not, suggesting different modes of mutagenesis between nuclear exonic mutations and mtDNA mutations in DLBCL. We also observed preferential accumulation of C > T and A > G transitions on the mitochondrial DNA heavy strand among both somatic mutations and private constitutional variants is consistent with studies of age-related mitochondrial somatic mutations in normal human tissues^29,32^ and it has been suggested that this strand bias may be introduced through an mtDNA replication-associated process^14,32^. Mitochondrial DNA replication starts at the H-strand origin of replication and uses the parental L-strand as a template; the parental H-strand remains single-stranded until the L-strand origin is reached^33^. Spontaneous deamination of C to U (retained as T) and A to hypoxanthine (retained as G), occurs frequently in human mtDNA^34^ and the single-stranded parental H-strand is even more susceptible to these deamination events^35^. The strand asymmetry we observed among both mitochondrial somatic mutations and private constitutional variants (Fig. 3b) lends support to this endogenous replication-associated model of mtDNA mutagenesis.

This model of mtDNA mutagenesis explains how C and A became minority nucleotides on the H-Strand over the course of mtDNA evolution and implies that the remaining C and A nucleotides on the H-strand are more likely to be important for mitochondrial function and consequently retained through purifying selection. This implies that C > T and A > G transitions on the H-strand generated primarily from an mtDNA replication-associated mechanism are more likely to be functionally consequential than other transitions. Among somatic mutations in DLBCL, which do not face strong selective pressures, this is the case: compared to C > T and A > G transitions on the L-strand, C > T and A > G transitions on the H-strand carried a higher proportion of amino acid changes as well as a higher mean pathogenicity score. In contrast, private constitutional variants are subject to purifying selection and consequently do not show any notable differences in the functional consequences of C > T and A > G transitions across strands (Fig. 4). This model of mutagenesis, together with the difference in selective pressures, would also explain why the extent of mutational asymmetry observed among mtDNA somatic mutations in DLBCL was greater than that of the constitutional private variants (Fig. 3b).

In contrast with Larman *et al*.’s study in AML and other cancers, we do not observe any strong indication of positive selection on mtDNA somatic mutations^20^. Other studies have observed purifying selection in other cancer types^14,21,23^, but we did not find evidence for purifying selection among mtDNA somatic mutations in DLBCL. Rather, our data suggest that mtDNA somatic mutations in DLBCL are generally selectively neutral and are tolerated by the tumour. We found that mtDNA variants in both normal and tumour tissue are generated predominantly through endogenous mtDNA replication-associated mechanisms that are distinct from nuclear mutagenesis and receive minimal contribution from ROS damage. These mechanisms target minority nucleotides and preferentially generate functionally consequential variants that face purifying selection in normal tissue but only neutral drift in DLBCL. Given this, we conclude that mtDNA somatic mutations are selectively neutral in DLBCL despite being more likely to compromise mitochondrial function, suggesting that mtDNA encoded mitochondrial function may not play an important role in promoting lymphomagenesis.

## Methods

### Samples and Data Access

Research ethics board approval was obtained from the Simon Fraser University Research Ethics Board and the joint University of British Columbia – BC Cancer Agency Clinical Research Ethics Board. Eighty WGS BAM files constituting matched tumour-normal samples of 40 patients from the CGCI were obtained through dbGaP (www.ncbi.nlm.nih.gov/gap)^36^. The WGS data (accession number phs000532.v6.p2) was originally generated at the Canada’s Michael Smith Genome Sciences Centre at the BC Cancer Agency using the Illumina HiSeq platform and aligned to the human hg18 reference sequence^37^.

### Variant Calling

The MitoSeek tool^38^ was revised (Supplementary Methods) and used to extract mitochondrial DNA reads from WGS BAM files of 40 tumour-normal pairs, convert the mitochondrial genome coordinates from the hg18 reference to the revised Cambridge Reference Sequence (rCRS) and conduct initial variant calling. The following conditions were applied for each position: minimum base alignment quality (BAQ) ≥20, minimum mapping quality (MQ) ≥20, minimum read depth ≥100, variant allele fraction (VAF) ≥0.02, variant allele count (VAC) ≥10 and for which the 99.999% confidence interval of the variant allele count (assuming a binomial distribution) did not overlap with zero. For somatic mutations, all filters remained the same, except that the difference in variant alleles between the tumour and normal was required to be ≥10 in allele count and ≥0.02 in allele fraction and the 99.999% confidence interval of the variant allele count under a binomial distribution did not overlap with the corresponding VAF in the normal. Variants reported by MitoSeek were further filtered for strand bias, in which variants were eliminated if they did not have ≥5 variant alleles called on each strand.

In analyzing mutational processes in normal tissue, we identified “private” constitutional variants, variants that are neither common in the population nor evolutionarily informative^39–41^. To identify these variants, we first used HaploGrep to determine the haplogroup of each sample^39^. From the constitutional variants, we removed variants that were accounted for by the sample haplogroup (global variants) as well as mutational super hot spots (A16182C, A16183C, T16519C), which are polymorphisms that are not haplogroup associated but occur very frequently in the general population^39^. The remaining constitutional variants were considered private.

### Variant Annotation

MutPred^17^ was used to predict the pathogenicity and functional consequences of non-synonymous variants present within the set of private constitutional variants and somatic mutations. The GenBank frequency of each variant was determined using MitoMaster^42^. At the time of this analysis, GenBank consisted of 30589 mitochondrial genomes.

### Mutational Spectrum

Nuclear variants were obtained from the supporting information of the published whole genome sequence analysis of the 40 patients^37^. Genomic coordinates were converted from hg18 to hg19 using the UCSC Liftover tool^43^; 3419 of 3424 variants were successfully converted. The compiled list of variants consisting of nuclear somatic mutations, mitochondrial somatic mutations and mitochondrial private constitutional variants were imported into R and mutation spectrum plots with consideration to 5′ and 3′ nucleotide context were generated using the SomaticSignatures package^44^.

### Statistical Analysis and Data Visualization

All statistical analysis was performed using R. Analysis of variant distributions across functional regions of the mitochondrial genome was performed using a chi-square test of homogeneity, analyses of heteroplasmic changes and relative copy number were performed using a paired t-test and analysis of treatment status was performed with a two-sample t-test. All p values are two-tailed. Tables were generated using Microsoft Excel; figures were generated using R, Circos^18^ and Inkscape.

### Accession number

The CGCI Non-Hodgkin Lymphoma – Diffuse Large B-Cell Lymphoma project dataset is available through dbGaP (www.ncbi.nlm.nih.gov/gap); accession number phs000532.v6.p2.

## Electronic supplementary material

Supplementary Information
