# Supplementary material for: Somatic Mitochondrial DNA Mutations in Diffuse Large B-Cell Lymphoma
Source: Sci Rep. 2018 Feb 26;8:3623. doi: 10.1038/s41598-018-21844-6 (PMC5827201; doi:10.1038/s41598-018-21844-6)
Supplement: Supplementary file 1 — Supplementary Information [file 41598_2018_21844_MOESM1_ESM.docx]

**Supplementary information**

**Somatic Mitochondrial DNA Mutations in Diffuse Large B-Cell Lymphoma**

Andy G.X. Zeng, Andy C.Y. Leung, and Angela R. Brooks-Wilson

**Supplementary Methods**

MitoSeek, a bioinformatics pipeline for extracting mtDNA information from genome sequencing data ^1^, was utilized to extract mitochondrial DNA reads from BAM files contasining the WGS data and detect heteroplasmic and homoplasmic variants. MitoSeek is an open-source tool written primarily in Perl that is capable of mitochondrial genome extraction from WES or WGS BAM files, heteroplasmic variant reporting, and somatic mutation reporting.

MitoSeek reports only the heteroplasmic fraction (the proportion of reads supporting the minor allele at a given locus) and does not determine whether an allele is the reference or the variant for any given position. MitoSeek also does not report homoplasmic variants and does not tolerate any level of noise in the basecall of the normal tissue for somatic mutation calling, requiring that all basecalls in the normal must support the major allele. Further, MitoSeek uses the universal genetic code instead of the mitochondrial genetic code in its amino acid annotations for each variant.

We revised the MitoSeek tool to address these issues. We integrated the use of the reference sequence in variant calling such that variant allele fractions are calculated (rather than heteroplasmic fractions), expanding the function of the tool from heteroplasmy detection alone to include both variant calling against a reference and homoplasmy detection. While MitoSeek tolerates the input of both rCRS (NC_012920) and Yoruba reference (NC_001807, used for chrM in hg18 and hg19) aligned BAM files, we revised the tool to ensure that all positions are converted to rCRS prior to variant calling in the revised version of the tool, given that variant reporting relative to the rCRS is the standard within the literature. Positions that surpassed both the variant allele fraction (VAF) and variant allele count (VAC) threshold were reported as heteroplasmic for that variant, while positions that did not meet this threshold were reported as either homoplasmic reference or homoplasmic variant depending on whether their predominant allele matched the reference allele. This approach was also utilized in the detection of somatic mutations and heteroplasmic changes. For matched tumour-normal samples, all positions in which the variant allele was classified as homoplasmic in the normal tissue were used to assess somatic mutations, while all positions in which the variant allele was classified as heteroplasmic in the normal tissue were used to assess heteroplasmic changes. As with heteroplasmy calling, variant allele differences that surpassed specified allele count and allele fraction thresholds were reported as somatic mutations or heteroplasmic changes. The variant annotation script was also revised to use the mitochondrial genetic code instead of the universal genetic code. Table S[1](#mitoseek_revisions) summarizes the revisions made to MitoSeek.

**Table S1 - Revisions made to MitoSeek**

| **Function** | **Feature** | **Original MitoSeek** | **Revised MitoSeek** |
| --- | --- | --- | --- |
| **Constitutional Variants** | Detects constitutional  Heteroplasmies | Yes | Yes |
|  | Detects constitutional  Homoplasmies | No | Yes |
|  | Calls variants against  a reference (rCRS) | No | Yes |
| **Somatic Mutations** | Detects Somatic  Mutations | Yes | Yes |
|  | Tolerates noise in  Normal basecall | No | Yes |
| **Heteroplasmic Changes** | Detects Heteroplasmic  Changes | No | Yes |
| **Variant Annotation** | Annotates Variants (gene, associated diseases, amino acid change) | Yes | Yes |
|  | Uses mitochondrial  genetic code | No | Yes |

rCRS = revised Cambridge Reference Sequence

We evaluated the MitoSeek revisions by using the tool to call variants on sample genomes from the 1000 Genomes Project and assessed the variant calling efficacy by using the variant calls from 1000 Genomes as a benchmark. We downloaded 30 random WGS BAM files from the 1000 Genomes Project database (http://www.1000genomes.org/), and used the revised MitoSeek tool to extract mtDNA reads and conduct variant calling, with a minimum depth threshold of 50 reads, VAF threshold of 0.05 and a VAC threshold of 10 reads for each position. The results were then compared to the variant call format (VCF) file provided by the 1000 Genomes Project Consortium.

The VCF file from the 1000 Genomes project reported 955 variants across the 30 mitochondrial genomes. While 19 of the variants called by 1000 Genomes did not meet the minimum depth threshold of 50 reads set for mtDNA variant calling using our tool, the remaining 936 variants that passed the depth threshold were all reported by the revised version of MitoSeek. One variant (C16188T, sample NA18612) was detected by revised MitoSeek but not reported in the variant calls provided by 1000 Genomes. We explored this discrepancy by inspecting the sample base call file: we found the position to have a sequence depth of 2496 reads, with 2484 reads in support of the variant (T) allele, confirming our categorization as a homoplasmic constitutional variant. The original version of MitoSeek did not detect any homoplasmic variants using the same parameters. In addition to calling homoplasmic variants, the revised MitoSeek tool detected 34 heteroplasmies using a 0.05 variant allele frequency threshold.

To analyze the mutational spectra of 40 DLBCL tumour-normal pairs, we made substantial edits to MitoSeek ^1^. Our test results using 1000 Genomes data demonstrate the utility of the revised MitoSeek tool in detecting both heteroplasmic and homoplasmic mtDNA variants, thereby justifying its use for our analysis of mitochondrial genomes in DLBCL.

**References**

1. Guo, Y., Li, J., Li, C.-I., Shyr, Y. & Samuels, D. C. MitoSeek: extracting mitochondria information and performing high-throughput mitochondria sequencing analysis. *Bioinformatics* **29,** 1210–1 (2013).

**Supplementary Table S1 - Somatic mtDNA mutations in DLBCL**

| **Position** | **Depth** | **VAF** | **Substitution** | **Gene** | **Coding Function** | **Codon, Amino Acid Changes** |
| --- | --- | --- | --- | --- | --- | --- |
| 64 | 1127 | 0.533 | C > T |  |  |  |
| 152 | 785 | 0.518 | T > C |  |  |  |
| 458 | 1514 | 0.041 | C > A |  |  |  |
| 709 | 1155 | 0.152 | G > A | RNR1 |  |  |
| 741 | 1783 | 0.059 | A > G | RNR1 |  |  |
| 1134 | 1148 | 0.774 | G > A | RNR1 |  |  |
| 1227 | 1622 | 0.158 | G > T | RNR1 |  |  |
| 1255 | 1969 | 0.027 | T > C | RNR1 |  |  |
| 1719 | 1810 | 0.831 | G > A | RNR2 |  |  |
| 1970 | 909 | 0.054 | G > A | RNR2 |  |  |
| 2407 | 1307 | 0.49 | T > C | RNR2 |  |  |
| 2510 | 2013 | 0.737 | T > C | RNR2 |  |  |
| 2555 | 2618 | 0.928 | C > T | RNR2 |  |  |
| 2593 | 2186 | 0.609 | G > A | RNR2 |  |  |
| 2732 | 881 | 0.093 | G > A | RNR2 |  |  |
| 2959 | 1920 | 0.027 | G > A | RNR2 |  |  |
| 3497 | 1638 | 0.024 | C > T | ND1 | non-synonymous | 3496-3498:GCC->GTC:A->V |
| 3506 | 1386 | 0.335 | C > T | ND1 | non-synonymous | 3505-3507:ACC->ATC:T->I |
| 3693 | 2085 | 0.186 | G > A | ND1 | synonymous | 3691-3693:CTG->CTA:L->L |
| 4007 | 1504 | 0.106 | T > C | ND1 | non-synonymous | 4006-4008:ATA->ACA:M->T |
| 4011 | 4464 | 0.641 | C > T | ND1 | synonymous | 4009-4011:AAC->AAT:N->N |
| 4047 | 1521 | 0.424 | T > C | ND1 | synonymous | 4045-4047:TAT->TAC:Y->Y |
| 4690 | 1334 | 0.445 | T > C | ND2 | non-synonymous | 4689-4691:ATC->ACC:I->T |
| 4973 | 1951 | 0.034 | T > C | ND2 | synonymous | 4971-4973:GGT->GGC:G->G |
| 5203 | 1011 | 0.043 | C > T | ND2 | non-synonymous | 5202-5204:CCA->CTA:P->L |
| 5244 | 1045 | 0.15 | G > A | ND2 | non-synonymous | 5244-5246:GGC->AGC:G->S |
| 5614 | 593 | 0.059 | C > A | TRNA |  |  |
| 6016 | 1002 | 0.079 | G > A | COX1 | non-synonymous | 6015-6017:CGA->CAA:R->Q |
| 6023 | 1012 | 0.339 | G > A | COX1 | synonymous | 6021-6023:GAG->GAA:E->E |
| 6113 | 1283 | 0.949 | A > G | COX1 | synonymous | 6111-6113:GTA->GTG:V->V |
| 6290 | 797 | 0.274 | C > T | COX1 | synonymous | 6288-6290:TAC->TAT:Y->Y |
| 6978 | 2537 | 0.581 | G > A | COX1 | non-synonymous | 6978-6980:GCA->ACA:A->T |
| 7010 | 3688 | 0.047 | C > A | COX1 | non-synonymous | 7008-7010:GAC->GAA:D->E |
| 7236 | 2581 | 0.038 | G > A | COX1 | non-synonymous | 7236-7238:GAT->AAT:D->N |
| 7337 | 2161 | 0.606 | G > A | COX1 | synonymous | 7335-7337:TCG->TCA:S->S |
| 7501 | 3440 | 0.063 | T > C | TRNS1 |  |  |
| 7898 | 2065 | 0.819 | T > C | COX2 | non-synonymous | 7898-7900:TAC->CAC:Y->H |
| 8557 | 1678 | 0.896 | G > A | ATP6 | non-synonymous | 8557-8559:GCC->ACC:A->T |
| 8881 | 1429 | 0.146 | T > C | ATP6 | non-synonymous | 8881-8883:TCT->CCT:S->P |
| 8902 | 1857 | 0.142 | G > A | ATP6 | non-synonymous | 8902-8904:GCC->ACC:A->T |
| 8989 | 2921 | 0.805 | G > A | ATP6 | non-synonymous | 8989-8991:GCC->ACC:A->T |
| 9225 | 3272 | 0.1 | G > A | COX3 | non-synonymous | 9225-9227:GCC->ACC:A->T |
| 9233 | 1654 | 0.499 | T > C | COX3 | synonymous | 9231-9233:CAT->CAC:H->H |
| 9554 | 2474 | 0.268 | G > A | COX3 | synonymous | 9552-9554:TGG->TGA:W->W |
| 9786 | 1085 | 0.054 | G > A | COX3 | non-synonymous | 9786-9788:GGC->AGC:G->S |
| 10589 | 2812 | 0.919 | G > A | ND4L | synonymous | 10587-10589:CTG->CTA:L->L |
| 11040 | 1874 | 0.099 | T > C | ND4 | non-synonymous | 11039-11041:CTC->CCC:L->P |
| 11166 | 2041 | 0.179 | G > A | ND4 | stopgain | 11165-11167:TGA->TAA:W->STOP |
| 11634 | 1977 | 0.042 | G > A | ND4 | non-synonymous | 11633-11635:AGC->AAC:S->N |
| 11711 | 3402 | 0.035 | G > A | ND4 | non-synonymous | 11711-11713:GCC->ACC:A->T |
| 11711 | 1644 | 0.053 | G > A | ND4 | non-synonymous | 11711-11713:GCC->ACC:A->T |
| 11742 | 5446 | 0.228 | G > A | ND4 | non-synonymous | 11741-11743:TGC->TAC:C->Y |
| 12611 | 2820 | 0.78 | T > C | ND5 | non-synonymous | 12610-12612:GTA->GCA:V->A |
| 13020 | 1181 | 0.087 | T > C | ND5 | synonymous | 13018-13020:GGT->GGC:G->G |
| 13920 | 2097 | 0.716 | C > A | ND5 | non-synonymous | 13918-13920:TTC->TTA:F->L |
| 13921 | 2889 | 0.929 | T > C | ND5 | non-synonymous | 13921-13923:TAC->CAC:Y->H |
| 14698 | 3572 | 0.401 | G > A | TRNE |  |  |
| 15535 | 3991 | 0.059 | C > T | CYTB | synonymous | 15533-15535:AAC->AAT:N->N |
| 15884 | 2203 | 0.765 | G > A | CYTB | non-synonymous | 15884-15886:GCC->ACC:A->T |
| 16295 | 1238 | 0.781 | C > T |  |  |  |

VAF = Variant Allele Fraction
